# Relative Telomere Length and Telomerase Reverse Transcriptase (TERT) Expression Are Associated with Age in Almond (*Prunus dulcis* [Mill.] D.A.Webb)

**DOI:** 10.3390/plants10020189

**Published:** 2021-01-20

**Authors:** Katherine M. D'Amico-Willman, Elizabeth S. Anderson, Thomas M. Gradziel, Jonathan Fresnedo-Ramírez

**Affiliations:** 1 Department of Horticulture and Crop Science, Ohio Agricultural Research and Development Center, The Ohio State University, Wooster, OH 446911, USA; damico-willman.1@osu.edu; 2 Center for Applied Plant Sciences, The Ohio State University, Columbus, OH 432102, USA; 3 Department of Biology, College of Wooster, Wooster, OH 44691, USA; anderson.3839@osu.edu; 4 Department of Plant Sciences, University of California, Davis, CA 95616, USA; tmgradziel@ucdavis.edu

**Keywords:** perennial, plant aging, biomarker, telomerase

## Abstract

While all organisms age, our understanding of how aging occurs varies among species. The aging process in perennial plants is not well-defined, yet can have implications on production and yield of valuable fruit and nut crops. Almond exhibits an age-related disorder known as non-infectious bud failure (BF) that affects vegetative bud development, indirectly affecting kernel yield. This species and disorder present an opportunity to address aging in a commercially relevant and vegetatively propagated perennial crop. The hypothesis tested in this study was that relative telomere length and/or telomerase reverse transcriptase (TERT) expression can serve as biomarkers of aging in almond. Relative telomere lengths and expression of TERT, a subunit of the enzyme telomerase, were measured via qPCR methods using bud and leaf samples collected from distinct age cohorts over a two-year period. Results from this work show a marginal but significant association between both relative telomere length and TERT expression, and age, suggesting that as almonds age, telomeres shorten and TERT expression decreases. This work provides information on potential biomarkers of perennial plant aging, contributing to our knowledge of this process. In addition, these results provide opportunities to address BF in almond breeding and nursery propagation.

## 1. Introduction

The current concept and study of aging is centered primarily around mammals with research focused on circumventing deleterious impacts on health [1,2]. However, all eukaryotic organisms exhibit signals of aging, resulting in the deterioration of key biological processes and subsequent decrease in health, performance, and fitness of individuals. Perennial plants represent a unique model to address the aging process and its impact since these species undergo cycles of dormancy and growth, and maintain the ability to reproduce for multiple years. The aging process of perennial plants is relevant due to the longevity and economic importance of perennial crops such as fruit and nut trees [3,4,5]. Individual trees can remain productive in orchards for decades; however, aging in plants and its implications for growth and reproduction are neglected areas of research with potential consequences for production, management, conservation, and breeding.

The lack of understanding of aging in perennials is partly due to the complexity in measuring and conceptualizing age in perennial plant species since chronologic and ontogenetic age are inversely related (i.e., newly emerged tissues are the youngest chronologically but the oldest ontogenetically) [6]. Chronologic age can be defined as the amount of time since tissue/organ formation (e.g., human skin cells replenish every few days, meaning each cell is typically a day or two days old), while ontogenetic age refers more to developmental time and allows for the accumulation of mutations or chromosomal alterations (e.g., two-day old skin cells at age six compared to two-day old skin cells at age 60). Agriculturally relevant perennials are often vegetatively propagated (i.e., cloned), blurring the distinction between ontogenetic and chronologic age, and tend to be grown under intensive management. The difficulty in determining age in perennials creates a need to identify biomarkers in these species that enable ontogenetic age estimation. 

**Figure 1:**
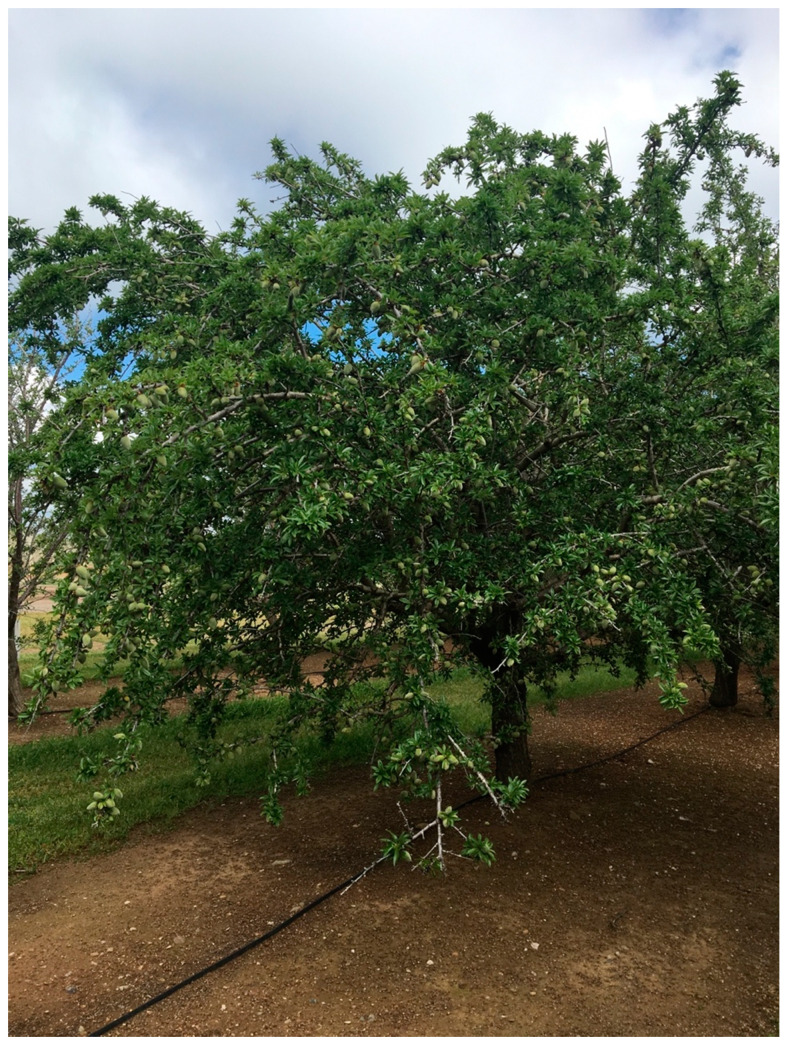
Image of almond cultivar Nonpareil (photo taken by K. D’Amico-Willman in May 2018).

Almond (*Prunus dulcis* [Mill.] D.A.Webb; Figure 1) is an economically relevant, Rosaceous crop, subject to intense horticultural management to maintain maximum nut production. In California, the almond industry is estimated to contribute ~$11 billion to the state’s gross domestic product annually [7]. Top-producing almond cultivars, some of which were seedlings first obtained more than 100 years ago, are produced for commercial orchards via vegetative propagation [8,9]. As orchards age (after 20–25 years), trees are replaced with “new” clones, typically of the same cultivar, to maintain high levels of production and homogeneity in quality [8].

Almond exhibits an age-related disorder known as non-infectious bud-failure (BF) affecting vegetative bud development in the spring [8,10]. Genotypes exhibiting this disorder show characteristic dieback at the top of the canopy, and severe levels of BF can result in up to 50% yield loss [11]. Empirical evidence shows BF is associated with age [12]; however, as almonds are produced primarily through vegetative propagation rather than by seed, their true ontogenetic age and thus susceptibility to BF can be difficult to assess [8]. Biomarkers indicative of age would be valuable to growers, breeders, and producers to screen germplasm. Thus, the almond represents a potential model species for the study of aging in perennials due to its economic relevance, the abundance of available germplasm and breeding programs, and the exhibition of an age-related disorder. 

Several biomarkers of aging have been studied in animals including protein glycation [13], DNA methylation [14], and telomere length [2,15,16]. Telomeres are nucleoproteins that cap the end of chromosomes, preventing premature instability of genomic material and cellular senescence [17]. Telomeres tend to shorten over mitotic cellular divisions due to decreased levels of telomerase, an enzyme that supports telomere replication during the S-phase of the cell cycle [18]. This progressive shortening is proposed as a marker of aging in mammalian cells and is linked to physiological deterioration and some age-related disorders [2,17,19]. Given that telomerase activity modulates telomere length, expression of genes involved in the telomerase biosynthetic pathway could also serve as biomarkers for aging [20,21,22,23,24]. Telomerase reverse transcriptase (TERT) is the catalytic subunit of the telomerase enzyme [25] and the RNA subunit functions as the template for reverse transcription [26]. Expression of TERT is shown to affect telomerase activity [27,28]. 

This study tests the hypothesis that relative telomere length and TERT expression in almond are associated with ontogenetic age and can thus be used to differentiate age cohorts and serve as biomarkers of aging in this species. Both relative telomere length and TERT expression show promise as diagnostic biomarkers since they can be measured in a high-throughput manner by applying a variety of informative methods [29,30,31]. These approaches build on previous research examining the relationship between telomere lengths and age in perennial plants [32,33,34,35]. The goal of this work is to advance our understanding and provide a model for the study of aging and its implications in perennial plant species.

## 2. Results

### 2.1. Association of Relative Telomere Length and Age in Almond

**Figure 2:**
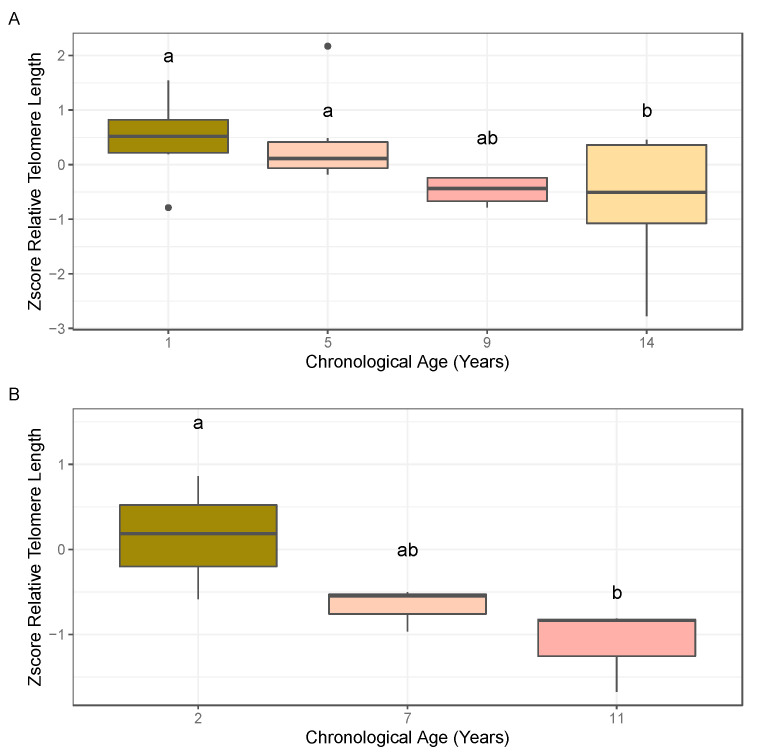
Boxplots depicting the calculated z-score of the T/S ratio for leaf samples within the age cohorts tested. (**A**) Age cohort collected in 2018. (**B**) Age cohort collected in 2019. Significant differences in z-scores between age cohorts based on ANOVA followed by post hoc Fisher’s least significant difference (LSD) (α = 0.1) are denoted by letter groupings where differing letters indicate significant differences following means separation analysis (ANOVA 2018 *p*-value = 0.1077; ANOVA 2019 *p*-value = 0.06548). Bold dots represent outliers within each age cohort.

**Figure 3:**
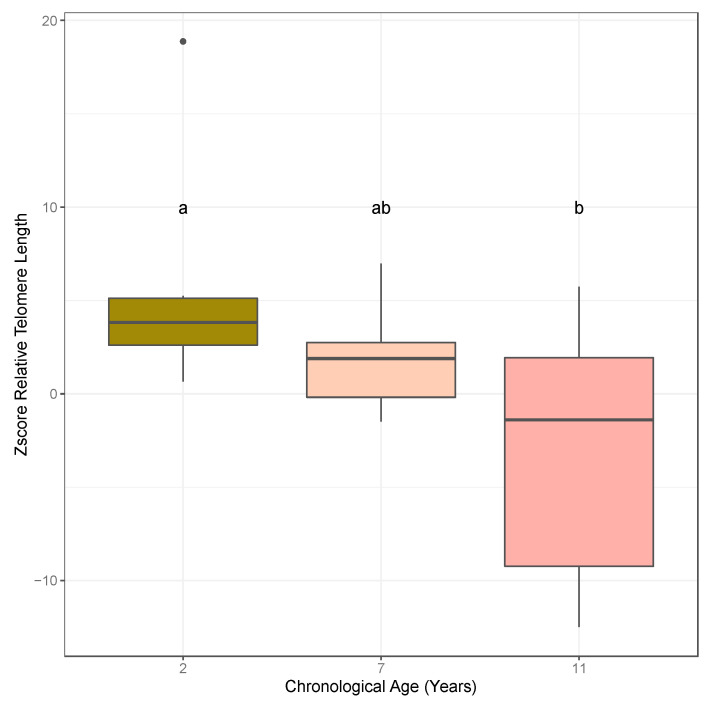
Boxplot depicting calculated z-score for the T/S ratio for bud samples within the age cohorts collected in 2019. Significant differences in z-scores between ages cohorts based on ANOVA followed by post hoc Fisher’s LSD (α = 0.05) are denoted by letter groupings where differing letters indicate significant differences following means separation analysis (ANOVA *p*-value = 0.067). Bold dots represent outliers within each age cohort.

Relative telomere lengths were generated for the almond individuals within each of the age cohorts collected in 2018 (1, 5, 9, and 14 years) using leaf tissue and in 2019 (2, 7, and 11 years old) using leaf and bud tissue following the monochrome multiplex quantitative PCR (MMQPCR) approach. Normality of residuals and homogeneity of variance of relative telomere lengths were confirmed using Shapiro–Wilks (2018: *p*-value = 0.2578, *n* = 4–6; 2019 (leaf): *p*-value = 0.4682, *n* = 3; 2019 (bud): *p*-value = 0.0402, *n* = 5–7) and Bartlett (2018: *p*-value = 0.1408; 2019 (leaf): *p*-value = 0.4613; 2019 (bud): *p*-value = 0.1076) tests. ANOVA results based on leaf tissue analysis for the linear model, z-score ~ age, were marginally significant in both 2018 and 2019, and subsequent post hoc Fisher’s least significant difference (LSD) and pairwise *t*-tests revealed significant differences between ages 1 and 14 years and 5 and 14 years (Figure 2A) in the 2018 cohorts, and between ages 2 and 11 years old (Figure 2B) in the 2019 cohorts. The ANOVA result based on the bud tissue analysis for the linear model, z-score ~ age, was significant at alpha = 0.1, and subsequent post hoc Fisher’s LSD and pairwise *t*-tests showed significant differences between ages 2 and 11 years old (Figure 3). Both bud and leaf tissue showed similar patterns in decreased relative telomere length with increased age.

### 2.2. TERT Gene Expression Patterns Associated with Age in Almond

**Figure 4:**
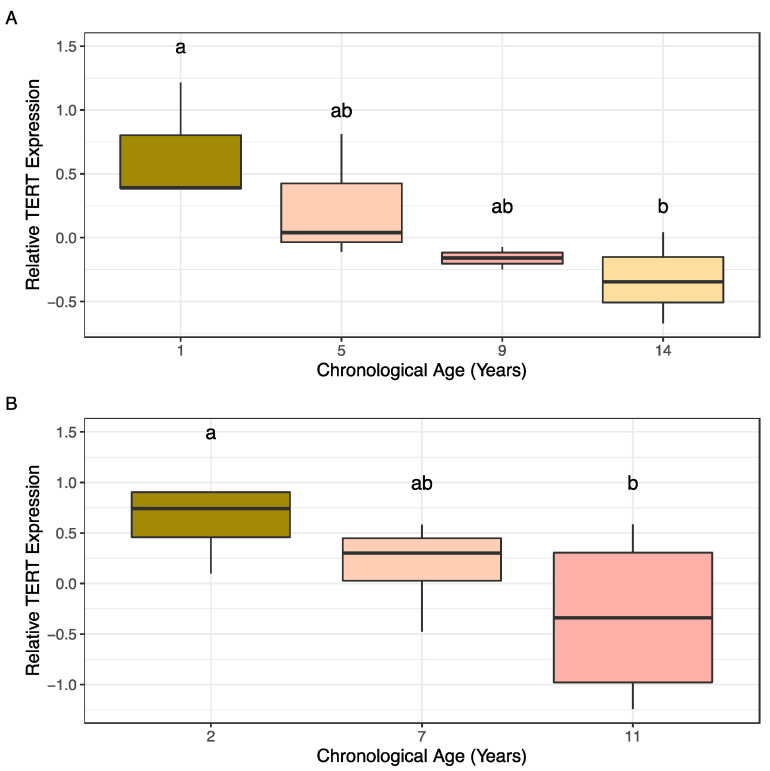
Normalized expression of TERT for almond samples within the age cohorts test. (**A**) Age cohort collected in 2018. (**B**) Age cohort collected in 2019. Significant differences in relative expression between age cohorts based on ANOVA followed by post hoc Tukey’s HSD (alpha = 0.1) are denoted by the letter groupings where differing letters indicate significant differences following means separation analysis (ANOVA 2018 *p*-value = 0.09087; ANOVA 2019 *p*-value = 0.1414).

Normalized expression of TERT was measured for almond samples among the age cohorts collected in 2018 and 2019 for this study using PP2A as the reference gene. Normality of residuals and homogeneity of variance were confirmed using Shapiro–Wilks (2018: *p*-value = 0.694, *n* = 2–3; 2019: *p*-value = 0.09456, *n* = 4) and Bartlett (2018: *p*-value = 0.6976; 2019: *p*-value = 0.3579) tests. ANOVA results comparing the average log (expression) values for each age cohort revealed significant differences between cohorts in both 2018 and 2019. Post hoc analysis with Tukey’s HSD revealed significant differences in TERT expression between ages 1 and 14 years old in the 2018 cohorts (Figure 4A) and between ages 2 and 11 years old in the 2019 age cohorts (Figure 4B).

## 3. Discussion

Almond, an economically valuable nut crop, exhibits an aging-related disorder known as non-infectious bud failure that negatively impacts vegetative development and, ultimately, yield. As a clonally propagated crop, tracking age and thus susceptibility to bud failure is difficult, making biomarkers of age a valuable resource to circumvent the impacts of aging-related disorders in almond germplasm. Relative telomere length is used as a biomarker of age and development of age-related disorders in mammals, but the association between relative telomere length and age in plants is not well-defined [17,26]. Over mitotic cell divisions, telomeres eventually reach a critical minimum length at which point the cell senesces and dies due to genome instability resulting from single stranded DNA at the ends of chromosomes [36]. Plant chromosomes also contain telomeres with similar functions. While the relationship between telomeres and the aging process is not as clearly defined in plants as in animals, previous work shows associations between relative telomere length and various stages of plant development [17,26,37] suggesting relative telomere length could be a suitable biomarker of age in plants. Additionally, telomerase activity modulates telomere length, and thus expression of genes involved in telomerase synthesis such as TERT could also serve as biomarkers of age. In Arabidopsis, increased TERT expression is linked to proportional increases in telomerase activity and telomere length [38,39], which are in turn linked to age. Since TERT is tied to telomere length in both plants and animals, its expression may also serve as an indicator of age in plants [17].

The present study tests the hypothesis that relative telomere length and/or TERT expression are associated with ontogenetic age in almond. To test this, a qPCR approach was utilized to measure relative telomere length and estimate TERT expression in sets of almond accessions of known chronological age. Leaf and bud samples were collected from three and four sets of age cohorts over two years to test for an association between relative telomere length and individual age using the MMQPCR method as well as between TERT expression and age using qRT-PCR.

### 3.1. Quantitative PCR Approaches Suggest an Association between Relative Telomere Length and Age in Almond Leaf and Bud Tissues

A pattern of decreased relative telomere length with increased age was shown utilizing MMQPCR and almond leaf and bud samples collected from different almond age cohorts in 2018 and 2019. The association demonstrated in this study adds to the growing body of knowledge regarding the complex relationship between telomere length and plant aging. Previous studies in both *Ginkgo biloba* and *Panax ginseng* showed a pattern of increased telomere length with increased age, suggesting plants do not follow the same patterns of telomere shortening as seen in mammals [34,35]. Work in apple (*Malus domestica*) and *Prunus yedoensis*, both members of the Rosaceae family like almond, show no change in telomere lengths with increased plant age over a five-year timespan [33]. In bristlecone pine (*Pinus longaeva*), a long-lived perennial gymnosperm, telomere lengths measured in needle and root tissues between 0–3500 years old showed a cyclical pattern of lengthening and shortening with age [32]. Further, when analyzing telomere length in relation to tissue differentiation, studies in both barley (*Hordeum vulgare*) and Scots pine (*Pinus sylvestris*) showed telomere shortening from embryo development to leaf or needle formation [40,41]. Similarly, in silver birch (*Betula pendula*), telomeres shorten when plant are grown in tissue culture conditions compared to those grown outdoors, suggesting abiotic stressors may also induce telomere shortening [42]. 

The results in almond suggest a pattern closest to what was observed in bristlecone pine where telomere lengths shorten and lengthen throughout an individual’s lifetime. This pattern could be unique to gymnosperms, however, and needs to be further characterized in angiosperms including Rosaceous species. While the commercial lifespan of productive almond clones is typically less than 30 years, almond seedlings can live more than 150 years [8]. In this study, the maximum age tested via qPCR was 14 years old, suggesting that a wider age range of trees and a larger sample size could produce a more refined model of telomere length patterns over time. 

Current almond cultivars may also be ontogenetically old. Nonpareil, the most relevant US cultivar representing ~40% of acreage, was first described almost 140 years ago and has been propagated by budding since [7,9]. The ontogenetic age of a cultivar may be a factor to consider in the onset of aging-related disorders like bud failure in almond. Additionally, it would be interesting to track the change in telomere length following clonal propagation (through budding) in which plants experience a rejuvenation process, reverting to a juvenile state for a short period of time [43]. Interestingly, both bud and leaf tissue showed similar patterns of decreased relative telomere length with increasing age in this study. The bud tissue utilized in this study was excised from stems containing the leaves that were also sampled. Based on their close proximity, it is possible that the telomere profile of the bud would be reflected in the associated leaf tissues. It would be useful to profile telomere lengths of buds throughout the tree to see if similar patterns of relative telomere length were obtained. It was further found that propagating almond from basal epicormic buds, potentially representing ontogenetically young meristematic tissue, seemed to alleviate BF in resulting clones [44]. Testing telomere lengths in epicormic tissues could present another avenue to both track aging in almond and develop biomarkers to predict BF potential in almond.

### 3.2. TERT Expression Measured by qRT-PCR Is Putatively Associated with Age in Almond Accessions

To test the hypothesis that TERT expression can serve as a biomarker of ontogenetic age in almond, expression patterns were tested in cohorts representing either three or four distinct ages over two years. Results from this work showed a consistent pattern of marginally significant, decreased expression with increased ontogenetic age. Telomerase was shown to be a modulator of longevity in humans and other mammals, but work describing telomerase patterns in plants is limited [20,22]. 

A comprehensive study examining telomerase protein activity in carrot (*Daucus carota*), cauliflower (*Brassica oleracea*), soybean (*Glycine max*), *Arabidopsis thaliana*, and rice (*Oryza sativa*) demonstrated that, like telomere lengths, protein activity tends to be highest in undifferentiated tissues like meristematic tissues and is lower in differentiated tissues such as leaves [20]. This result was supported by further work in barley and maize showing little activity in differentiated tissues [45]. These studies were all performed in annuals or biennials, however, suggesting that telomerase activity does in fact decrease with increased plant age in these crops. Work in perennials including bristlecone pine, *P. ginseng*, and *G. biloba* showed an association between telomerase activity and age, suggesting patterns unique to perennial plant species [32,34,46]. A study in almond could be performed using a wider age range and larger sample size to elucidate the effect of age on telomerase activity, similar to what was referenced above for telomere length measurements. Additionally, many of the studies performed in other plants examining patterns of telomerase activity focused on protein activity rather than gene expression. A future study will be necessary in almond to examine the telomerase protein activity, potentially by Western blot or other proteomics approaches, to corroborate the association between TERT expression and protein activity.

While a pattern was established in plants demonstrating a direct relationship between telomerase activity and telomere length, regulation of telomerase is still not well understood in the plant kingdom [20,28,37]. Interestingly, work in Arabidopsis has shown a link between DNA methylation and telomere length, suggesting that this epigenetic mark likely has a role in regulating telomere lengths potentially by modulating telomerase activity [47,48,49]. A study is ongoing in almond to analyze DNA methylation patterns in a set of almond accessions representing three distinct age cohorts to determine what, if any, impact age has on methylation profiles. 

Despite the limited age range and small sample size used in this study, a consistent pattern of both decreased relative telomere length and decreased TERT expression with increased age was observed over two years of sampling, regardless of whether the sample was taken from actively growing tissue like buds or a more static tissue in terms of cell division, like leaves. These results provide a basis for future study and exploration into the utility of relative telomere length measurement and/or TERT expression or telomerase activity as biomarkers of aging in almond. Developing a robust biomarker to track aging in almond, a primarily clonally propagated crop, would allow growers, producers, and breeders to screen germplasm to eliminate selections or clones with a high susceptibility to age-related disorders due to advanced ontogenetic age. 

## 4. Materials and Methods

### 4.1. Plant Material

Leaf samples for this study were collected in May 2018 and 2019 from almond breeding selections located at the Wolfskill Experimental Orchards (Almond Breeding Program, University of California—Davis, Winters, CA, USA). Leaf tissue was harvested from the upper canopy of a total of 36 unique individuals representing distinct age cohorts (Table 1). Vegetative buds were sampled in May 2019 from the upper canopy stem segments of the 18 unique individuals used for leaf sample collection (Table 1). Samples were immediately frozen on ice and stored at −20 °C until shipment overnight on dry ice to the Ohio Agricultural Research and Development Center (OARDC—Wooster, OH, USA). Samples were stored at −20 °C until processing, and all subsequent experimental procedures were conducted at the OARDC.

### 4.2. DNA and RNA Extraction

DNA was extracted from the leaf samples using the Omega E-Z 96^®^ Plant DNA Kit (Omega Bio-tek, Norcross, GA, USA) with slight modification. Briefly, 100 mg of leaf material was weighed in 2.0 mL tubes containing two 1.6 mm steel beads and kept frozen in liquid nitrogen. Samples were ground in a 2000 Geno/Grinder^®^ (SPEX SamplePrep, Metuchen, NJ, USA) in two 48-well cryo-blocks frozen in liquid nitrogen. Following a 65 °C incubation, samples were incubated on ice for 20 min, treated with 10 μL of RNase solution (2.5 μL RNase [Omega Bio-tek, Norcross, GA, USA] + 7.5 μL TE pH 8), equilibrated through addition of 150 μL equilibration buffer (3 M NaOH), incubated at room temperature for four minutes, and centrifuged at 4400 rpm for two minutes prior to the addition of SP3 buffer. Concentration and quality were analyzed using a NanoDrop™ 1000 spectrophotometer and a Qubit 4 Fluorometer with a dsDNA HS Assay Kit (ThermoFisher Scientific, Waltham, MA, USA).

DNA was extracted from bud samples following a modified version of the protocol outlined in Vilanova et al. [50]. Briefly, 5 buds from each sample were ground in a 2 mL microfuge tube with one 3.2 mm steel bead using a 2000 Geno/Grinder^®^ (SPEX^®^ SamplePrep, Metuchen, NJ, USA) set at 200 strokes per minute for 5 min. Finely ground tissues were added to 1 mL of extraction buffer (2% *w/v* CTAB; 2% *w/v* PVP-40; 20 mmol/L EDTA; 100 mmol/L Tris-HCl [pH 8.0]; 1.4 mol/L NaCl), 14 μL beta-mercaptoethanol, and 2 μL RNase (10 mg/mL). The solution was incubated at 65 °C for 30 min and on ice for 5 min followed by a phase separation with 700 μL chloroform:isoamyl alcohol (24:1). The aqueous phase (~800 μL) was recovered, and 480 μL binding buffer (2.5 mol/L NaCl; 20% *w/v* PEG 8000) was added followed by 720 μL 100% ice-cold ethanol. 

Silica matrix buffer was prepared by adding 10 g silicon dioxide to 50 mL ultra-pure water prior to incubation and centrifugation steps. Silica matrix buffer (20 μL) was added to each sample, and samples were gently mixed for 5 min. Samples were spun for 10 s and supernatant was removed. To resuspend the remaining mucilaginous material (but not the pellet), 500 μL cold 70% ethanol was used and supernatant was removed. Another 500 μL cold 70% ethanol was added to resuspend the silica pellet, the tubes were spun for 5 s, and the supernatant was removed. The pellet was allowed to dry at room temperature for 5 min and was resuspended in 100 μL elution buffer (10 mmol/L Tris HCl [pH 8.0]; 1 mmol/L EDTA [pH 8.0]) followed by a 5 min incubation at 65 °C. Samples were centrifuged at 14,000 rpm for 10 min at room temperature and 90 μL of supernatant was transferred to a new tube. DNA concentration was assessed by fluorometry using a Qubit™ 4 and Qubit™ 1X dsDNA HS Assay Kit (ThermoFisher Scientific, Waltham, MA, USA).

RNA was extracted from leaf tissue following the protocol outlined in Gambino et al. [51] with slight modifications. Briefly, leaf material was ground in liquid nitrogen using a mortar and pestle, and 150 mg of tissue was weighed into a 2.0 mL microfuge tube frozen in liquid nitrogen. To extract RNA, 900 μL CTAB extraction buffer (2% CTAB, 2.5% PVP-40, 2 mol/L NaCl, 100 mmol/L Tris-HCl pH 8.0, 25 mmol/L EDTA pH 8.0, 2% beta-mercaptoethanol added before use) was added to each tube and samples were incubated at 65 °C for ten minutes. Following incubation, two phase separations were performed using an equal volume of chloroform:isoamyl alcohol (24:1). RNA was precipitated in 3 mol/L lithium chloride and incubated on ice for 30 min, and samples were pelleted by centrifugation at 21,000× *g* for 15 min. Pellets were then resuspended in 500 μL pre-warmed SSTE buffer (10 mmol/L Tris-HCl pH 8.0, 1 mmol/L EDTA pH 8.0, 1% SDS, 1 mol/L NaCl) followed by a phase separation with an equal volume of chloroform:isoamyl alcohol (24:1). A final precipitation was performed using 0.7 volume chilled 100% isopropanol. RNA was pelleted and washed with 70% ethanol before being resuspended in 30 μL nuclease-free water. A DNase treatment was performed using DNA-*free*™ DNA Removal Kit (ThermoFisher Scientific) according to the manufacturer’s instructions. All materials used for extraction were nuclease-free and cleaned with RNaseZap™ RNase decontamination wipes (ThermoFisher Scientific) prior to use. All centrifugation steps were performed at 4 °C. RNA quality and concentration were assessed using a NanoDrop™ 1000 spectrophotometer and a Qubit 4 fluorometer with an RNA HS Assay Kit (ThermoFisher Scientific).

### 4.3. Monochrome Multiplex Quantitative PCR (MMQPCR) to Measure Relative Telomere Lengths

MMQPCR was conducted following the protocol outlined in Vaquero–Sedas and Vega–Palas [52] with minimal modifications. Primer sequences for genes used in this study are shown in Table 2, including primers for the single copy gene, PP2A, and for the telomere sequence [52,53]. Oligos were synthesized by MilliporeSigma (Burlington, MA, USA) and resuspended to a concentration of 100 μmol/L upon arrival. Standard curves were created for each primer pair by pooling six aliquots of DNA isolated from a single clone of the almond cultivar Nonpareil, and performing successive dilutions to 20 ng/μL, 10 ng/μL, 1 ng/μL, 0.5 ng/μL, and 0.25 ng/μL. Reactions were carried out in triplicate for each primer by concentration combination. 

Isolated DNA from the age cohort samples was diluted to 20 ng/μL. Multiplex reactions were carried out in sextuplicate for each replicate within the age cohorts in a 10 μL volume using QuantaBio PerfeCTa SYBR^®^ Green SuperMix (Quanta Biosciences, Beverly, MA, USA) (2×), forward and reverse primers (100 nmol/L each), and 20 ng template DNA according to the manufacturer’s instructions. Reactions were performed in a Bio Rad C1000 Touch Thermal Cycler (Bio Rad Laboratories, Hercules, CA, USA) using the following program: initial denaturation at 95 °C for 3 min followed by 2 cycles of incubation at 94 °C for 15 s and annealing at 49 °C for 15 s; telomere and PP2A amplicons were generated following 35 cycles at 95 °C for 30 s, 59 °C for 1 min, 72 °C for 30 s, 84 °C for 15 s, and 85 °C for 15 s; final incubation at 72 °C for 1 min. Melting curve analysis was performed at a temperature range of 74–85 °C for both primer pairs to ensure no non-specific amplification.

### 4.4. cDNA Synthesis and Quantitative Reverse Transcriptase PCR (qRT-PCR) to Measure Relative Expression of TERT

Reactions were carried out in a 20 μL volume using the Verso™ cDNA Synthesis Kit (ThermoFisher Scientific). One reaction was prepared for each age cohort sample according to the manufacturer’s instructions. Reactions were performed in an MJ Research PTC-200 thermal cycler using the following program: 42 °C for 30 min followed by 95 °C for 2 min. To quantify expression of TERT in age cohort individuals, qRT-PCR was performed in triplicate for each sample. The gene RPII from peach was used as a reference [54,55], and the sequence for the TERT gene was derived from the Texas genome (https://www.rosaceae.org/analysis/295) using the homologous peach gene sequence as a reference [56]. Primer sequences are shown in Table 2, and all oligos were synthesized by MilliporeSigma (Burlington, MA, USA) and resuspended to a concentration of 100 μmol/L upon arrival. 

To generate cDNA from the age cohort samples, 100 ng of RNA was used as input in the Verso cDNA Synthesis Kit (ThermoFisher Scientific) according to the manufacturer’s instructions. To test for relative expression of TERT, reactions were carried out in triplicate for each biological replicate within the age cohorts in a 10 μL volume using QuantaBio PerfeCTa SYBR^®^ Green SuperMix (Quanta Biosciences) (1×), forward and reverse primers (100 nmol/L), and cDNA (1 μL) according to the manufacturer’s instructions. Reactions were performed in Bio Rad C1000 Touch Thermal Cycler (Bio Rad Laboratories) using the following program: initial denaturation at 95 °C for 3 min followed by 40 cycles at 95 °C for 15 s and 55 °C for 45 s. Melt curves were generated at a temperature range of 74–85 °C for both primer pairs to ensure no non-specific amplification. 

### 4.5. Statistical Analysis

Using the standard curve generated with PP2A (S) and telomere (T) primers for a reference almond sample, relative T/S ratios were calculated for each individual sample based on Cq values for the telomere and PP2A products [52]. Z-scores were calculated from the T/S ratios as recommended in Verhulst [57] for each replicate within the age cohorts. Normality and homogeneity of variance were confirmed using Shapiro–Wilks and Bartlett tests. Analysis of variance (ANOVA) was performed for each age cohort followed by *post hoc* Fisher’s LSD and pairwise *t*-tests. Gene expression data were analyzed according to guidelines in Bustin et al. [58], first by normalizing *TERT* expression to that of the reference gene, *RPII*. Following normalization, data were log-transformed, and normality and homogeneity of variance were confirmed using Shapiro-Wilks and Bartlett tests. ANOVA was performed for each age cohort followed by post hoc analysis with Tukey’s HSD. Letter groupings indicate significant means separation following significant ANOVA results. Shared letters indicate that means did not significantly differ between groups, while different letters indicate a significant difference between means when comparing groups. All analyses were performed using R v. 3.6.1 and plots were generated using ggplot2 v. 3.3.0. Calculated T/S ratios, relative telomere lengths, relative TERT expression and log-transformed TERT expression as well as raw Cq values for each individual are listed in Supplementary File S1. All R code used to perform analyses is reported in Supplementary File S2. Analyses were performed using the Ohio Supercomputer Center resources [59]. 

## Figures and Tables

**Table 1:** Sampling scheme for 2018 and 2019 almond age cohort collections.

Sampling Year	Age (Years)	Leaf Sample Size	Bud Sample Size
2018	1	6	N/A
2018	5	6	N/A
2018	9	6	N/A
2018	14	6	N/A
2019	2	4	6
2019	7	4	7
2019	11	4	5

**Table 2:** Oligos used for all Monochrome Multiplex Quantitative PCR (MMQPCR) and quantitative reverse transcriptase PCR (qRT-PCR) studies.

Oligo Name	Oligo Sequence (5′–3′)
*PP2A Forward*	CGGCGGCGGGCGGCGCGGGCAGGATAGACATTGGAGGGTTCGGCTCGCAA
*PP2A Reverse*	CGGCGGCGGGCGGCGCGGGACCACTGCATGCAAAGGGACCCAAGCTTAT
*Telomere Forward*	CCCCGGTTTTGGGTTTTGGGTTTTGGGTTTTGGGT
*Telomere Reverse*	GGGGCCCTAATCCCTAATCCCTAATCCCTAATCCCT
*TERT Forward*	GCATCAGAGAAGGGTCAGATT
*TERT Reverse*	CTCTGGCTCCTTGAATCGTATAG
*RPII Forward*	TGAAGCATACACCTATGATGATGAAG
*RPII Reverse*	CTTTGACAGCACCAGTAGATTCC

## Data Availability

The data presented in this study are available in the Supplementary Materials section of this manuscript.

## Supplementary Materials

The following are available online at https://www.mdpi.com/2223-7747/10/2/189/s1, File S1: File containing raw data for each experiment including mean telomere length quantification and TERT expression analysis., File S2: R code used to perform all statistical analyses reported in this manuscript.
